# Molecular epidemiology and structural diversity of O101/O162 O-antigen variants among *Escherichia coli* bacteremia isolates

**DOI:** 10.1038/s41598-026-45688-7

**Published:** 2026-03-23

**Authors:** Eveline Weerdenburg, Mark de Been, Aldert Zomer, Wannisa Ritmahan, Joyce Lübbers, Alan B. Moran, Simone Nicolardi, Manfred Wuhrer, Neil Ravenscroft, Chakkumkal Anish, Jeroen Geurtsen, Michel Beurret

**Affiliations:** 1 Janssen Vaccines & Prevention B.V, Johnson & Johnson, Leiden, The Netherlands; 2 Department of Infectious Diseases and Immunology, Faculty of Veterinary Medicine, Utrecht University, Utrecht, The Netherlands; 3 Center for Proteomics and Metabolomics, Leiden University Medical Center, Leiden, The Netherlands; 4 Department of Chemistry, University of Cape Town, Rondebosch, South Africa; 5 Present Address: Vaccine & Immunetherapies Division, AstraZeneca, Gaithersburg, MD USA; 6 Present Address: Sanofi B.V, Amsterdam, The Netherlands

**Keywords:** Diseases, Microbiology

## Abstract

Most human invasive infections caused by extraintestinal pathogenic *Escherichia coli* (ExPEC) are associated with a limited number of O-serotypes and O-serogroups, including O101/O162. Here, we examined *E. coli* O101/O162 O-serogroup epidemiology among bacteremia isolates and performed genetic, biochemical and structural analyses. We demonstrate that the O101/O162 O-serogroup is globally widespread and highly associated with multidrug resistance (MDR). Dominant lineages belonged to clonal complex 10, including the high-risk clone ST167. Most O101/O162 ExPEC isolates contained an unusual O101 *rfb* locus previously identified as Onovel32, of which 30% harbored disruptions in the methyltransferase (MT) encoding gene. These disruptions resulted in alteration of the polysaccharide composition, and we identified two major O101 variants. Additionally, we found that an intact MT is a prerequisite for *O*-methylation of the polysaccharide, indicating terminal capping. Our data supports a model of a co-polymeric O101 O-antigen structure, which is unique for *E. coli*. Finally, we established that the two clinically relevant O101 O-antigen variant structures are highly immunogenic as polysaccharide-protein conjugates but differ in their ability to elicit opsonophagocytic antibodies. Altogether, our data provides important insights into O-serotype epidemiology and O-polysaccharide variation within *E. coli* O101/O162 isolates associated with bacteremia.

**Supplementary Information:**

The online version contains supplementary material available at 10.1038/s41598-026-45688-7.

## Introduction

Extraintestinal pathogenic *Escherichia coli* (ExPEC) is a major bacterial pathogen that is one of the most common causes of nosocomial and healthcare-associated infections. In adults, it is a leading cause of bacteremia, sepsis and subsequent hospitalization and sometimes death^1,2^. Additionally, ExPEC is one of the most common causative pathogens of urinary tract infections (UTI) and neonatal meningitis^3,4^. An important virulence factor of the pathogen is the O-antigen, the distal-end polysaccharide structure of lipopolysaccharide (LPS) that is located on the cell surface of *E. coli*. Among other functions, this structure plays an important role in serum resistance, promoting survival of the bacteria in the (human) host. Over 180 different O-antigen structures and substructures have been described for *E. coli*, forming the foundation of the antiserum-based O-serotyping scheme^5^. The O-antigens differ in monosaccharide composition and/or linkages within and between the individual repeat unit (RU) structures that constitute the polysaccharide chains. These structural differences are mainly determined by genetic variation in the O-antigen biosynthesis locus, known as *rfb*.

The majority of invasive *E. coli* infections are associated with a limited number of O-serotypes^6^. The surface-exposed O-antigen therefore forms a potential vaccine target that has been evaluated in several pre-clinical and clinical studies, although the development of an effective vaccine to prevent invasive *E. coli* disease (IED) remains challenging^7–11^. Many of the O-serotypes identified among clinical ExPEC bloodstream isolates show a stable prevalence over time. However, a rapid emergence of particular clones with selective advantages can occur as was exemplified by the global spread of the *E. coli* O25b-ST131 lineage among human ExPEC infections from the early 2000’s onwards^12^. This lineage is associated with multi-drug resistance (MDR) and has become the dominant cause of IED and UTI^6,13^. Epidemiological surveillance programs are important to detect the emergence and monitor the prevalence of high-risk ExPEC lineages associated with antimicrobial resistance (AMR) such as O25b-ST131. Interestingly, in a recent study we have found that the O101/O162 O-serogroup is commonly observed among MDR *E. coli* bloodstream isolates and shows a relatively high prevalence in South America, a region where AMR rates are generally relatively high^6^. O-serotype O101 is commonly associated with pathogenic *E. coli* strains causing UTI and bloodstream infections but also with those causing diarrheal disease in both humans and animals^14–17^. Several reports have described MDR and even extensively drug resistant (XDR) *E. coli* O101 strains^14,15^. This O-serotype has also been linked to the high-risk ST167 lineage associated with the *bla*NDM-5 determinant that provides resistance to last-resort carbapenem antibiotics^18,19^. Drug-resistant *E. coli* O101 strains seem to be well equipped to cause successful infections, and specific lineages may have the potential to follow the concerning trajectory of the pandemic O25b-ST131 clone.

On the polysaccharide level, the two individual O-serotypes O101 and O162 that are part of the *E. coli* O101/O162 O-serogroup are structurally and genetically related and show serological cross-reactivity^20^. O89 has been described to cluster with this group based on genetics. However, O89 is serologically distinct from O101 and O162^20^, and the reference strain of this O-serotype has been described as rough, meaning that it lacks O-antigen expression^5,21^. The O-antigen polysaccharides of the O101 and O162 O-serotypes have in common two different backbone structures with disaccharide RUs composed of *N*-acetylglucosamine (GlcNAc) and *N*-acetylgalactosamine (GalNAc) (PS1, →6)-α-D-Glc*p*NAc-(1→4)-α-D-Gal*p*NAc-(1→) or two GalNAc monosaccharides (PS2, →4)-β-D-Gal*p*NAc-(1→4)-α-D-Gal*p*NAc-(1→)^22,23^. Interestingly, the co-existence of these two distinct RU forms has been described in polysaccharides of O101 and O162 *E. coli* strains, which is unusual for this species^22^. O162 can be differentiated from O101 by an additional 4-deoxy-D-*arabino*-hexose (ara4dHex) side-branch linked to the PS1 or PS2 backbone structures. Based on the presence of a predicted methyltransferase (MT) enzyme encoded within the *rfb* locus of *E. coli* O101 and O162, the presence of a nonreducing terminal modification on the polysaccharide chain has been hypothesized, although this has not been proven experimentally^5^. Such modifications are common chain-length regulating features of other *E. coli* serotypes that, like O101 and O162, use the ABC-transporter pathway for O-antigen biosynthesis, including O8 and O9^24^.

Due to their structural and genetic similarities and serological cross-reactivity, O101 and O162 *E. coli* strains are difficult to distinguish. As the *rfb* loci of O101 and O162 are nearly identical with an overall DNA identity of 99.8%^5,20^, sequence-based genotyping tools that use the serotype-specific *wzx/wzy* and *wzm/wzt* genes are unable to differentiate O101 from O162 and additionally, sometimes report strains belonging to this O-serogroup as O89. Moreover, a report from 2016 described a new serotype Onovel32^25^, which appears to group together with O101, O162 and O89 on the genetic level. An identical *rfb* gene cluster was later designated as O89m^21^, and O89b^26^. As a result, different serotyping outcomes can be obtained for the same *E. coli* strains depending on the methods and references used, leading to an unclear picture of clinically relevant strains within the *E. coli* O101/O162 O-serogroup. This could potentially lead to underreporting and delayed detection of globally emerging high-risk *E. coli* O101/O162 lineages associated with MDR.

The *E. coli* surveillance studies we have conducted over the past years have provided us with a large collection of contemporary O101/O162 O-serogroup ExPEC bacteremia isolates^6,27,28^, which we further analyzed in the current study. Genetic and biochemical analyses revealed the dominance of two variants of O101 within this O-serogroup, which showed differences in polysaccharide composition upon structural characterization. Furthermore, we identified a single methyl-modification on the O-antigen polysaccharide chain of the main variant, suggesting the presence of an end cap at the nonreducing terminus. This modification was found to be dependent on the presence of an intact MT encoding gene that was disrupted in 30% of *E. coli* O101/O162 isolates. Finally, polysaccharide-protein conjugates generated from the two O101 variants showed differences in their potential to elicit an immune response that could facilitate opsonophagocytic killing of O101/O162 O-serogroup ExPEC strains. Our results give important insights into drug-resistant lineages and antigenic variants within *E. coli* O101/O162 strains causing bacteremia and demonstrate the immunological consequences of O-antigen variation.

## Results

### *E. coli* O101/O162 bacteremia isolates carrying Onovel32 *rfb* loci are globally widespread and associated with MDR

**Fig. 1:**
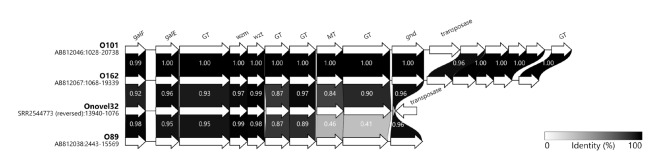
Alignment of *rfb* gene cluster sequences from reference loci of *E. coli* O101 (AB812046), O162 (AB812067), Onovel32 (SRR2544773) and O89 (AB812038). The *rfb* locus is located between *galF* and *gnd* genes. Directly downstream of *gnd*, O101 and O162 *rfb* loci contain a set of putative additional O-antigen biosynthesis genes hypothesized to modify the RU of O162 but not of O101 due to a transposon insertion in the latter^5^. Numbers indicate fraction of sequence identity at the protein level. GT: glycosyltransferase, MT: methyltransferase.

To determine the global prevalence of the O101/O162 O-serogroup among ExPEC isolates, we performed an O-genotype-based screening on a genome sequence dataset of 7439 *E. coli* bloodstream isolates collected globally from adult bacteremia patients in previous studies^6,27,28^. To this end, we used reference *rfb* loci of *E. coli* O101, O162, O89 and Onovel32, all previously described to be associated with this O-serogroup^5,20,25^. Although these loci have the same genetic organization, Onovel32 and O89 displayed substantial differences in sequence identity of the putative MT- and glycosyltransferase- (GT) encoding genes relative to O101 and O162 and to each other (Fig. 1). Based on a cutoff of > 99% sequence identity to the reference loci, we identified 191 *E. coli* strains in our collection containing an Onovel32 *rfb* locus, one strain containing an O162 *rfb* locus and no strains containing an O101 or O89 *rfb* locus. Surprisingly, these findings reveal that Onovel32 is the dominant *rfb* locus within the O101/O162 O-serogroup among *E. coli* bacteremia isolates. For 27 of the 192 identified *E. coli* O101/O162 isolates, agglutination O-serotyping was performed to confirm functional O101 or O162 O-antigen expression. Our results showed that most of the tested Onovel32 *rfb* containing strains were agglutination-positive for O101, although some strains showed a positive response for O162 (Table 1).

**Table 1:** Results from *rfb* locus sequence-based O-genotyping and phenotypic agglutination O-serotyping of 27 clinical *E. coli* O101/O162 blood isolates. Numbers indicate the number of isolates falling in each group.

		Agglutination O-serotype	
		O101	O162
O-genotype	O101	0	0
	O162	0	1
	Onovel32 (MT-)	19 (0)	7 (4)

**Fig. 2:**
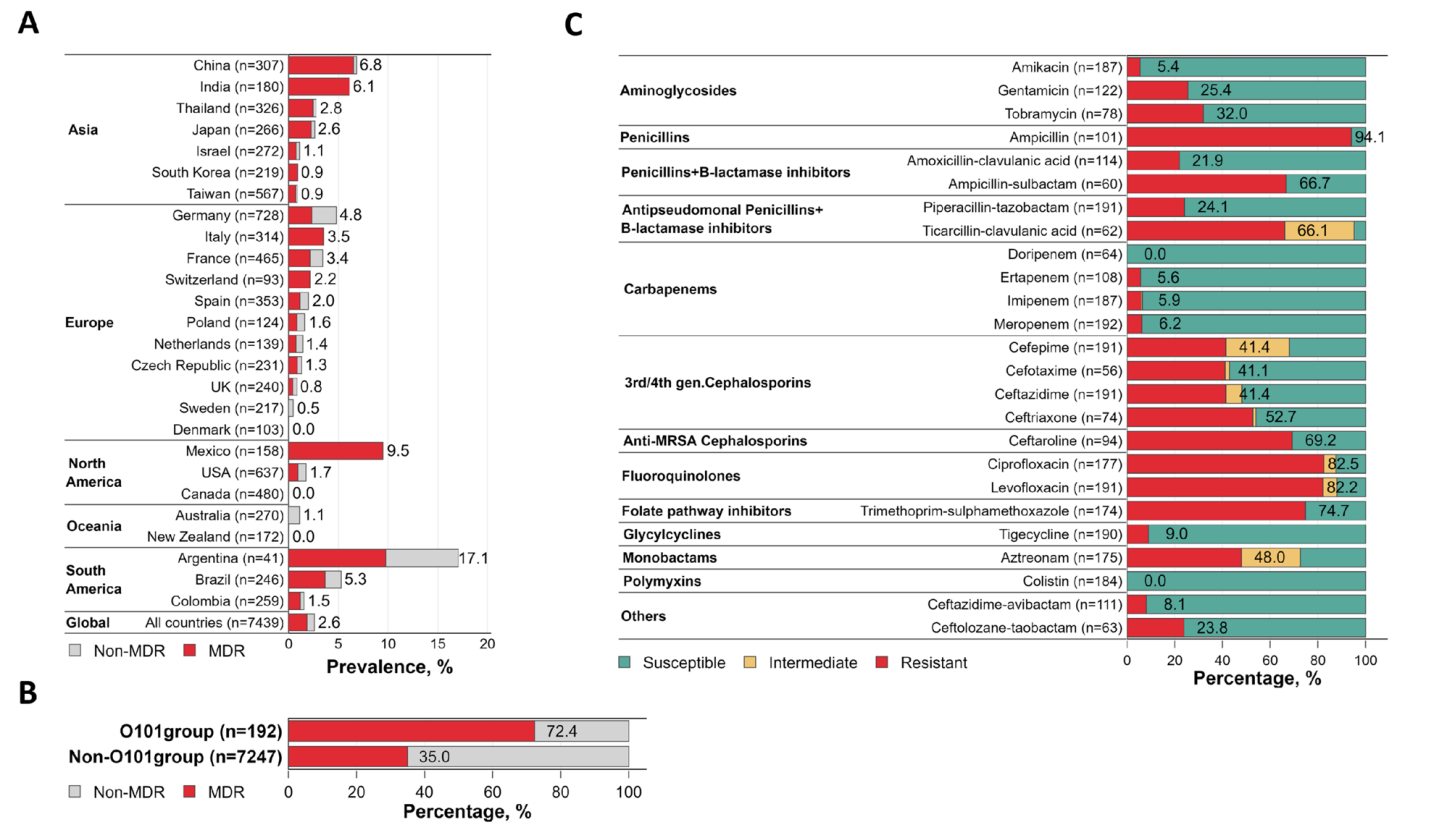
Prevalence and drug resistance of *E. coli* O101/O162 blood isolates. **(A)** Country-specific prevalence of *E. coli* O101/O162 bacteremia isolates. Red color indicates the level of MDR within the group of O101/O162 O-serogroup *E. coli* isolates for each country. Total number of available bacteremia isolates per country is indicated between brackets. Only countries that include 40 or more isolates are displayed. **(B)** Percentage of MDR among *E. coli* O101/O162 blood isolates in comparison with non-O101/O162 O-serotypes. **(C)** Percentage of *E. coli* O101/O162 isolates resistant to individual antibiotics within different classes of antimicrobial agents. The number of isolates tested for each drug is indicated between brackets.

We observed the presence of *E. coli* O101/O162 in 22 out of 30 countries represented in the global analysis set, distributed across different geographical regions and showing an overall global prevalence of 2.6% (Fig. 2A). The highest prevalence of the O101/O162 O-serogroup was observed in Argentina (7/41, 17.1%), followed by Mexico (15/158, 9.5%), China (21/307, 6.8%) and India (11/180, 6.1%). The overall degree of MDR among *E. coli* O101/O162 was 72.4%, which is considerably higher than the average degree of 35.0% MDR among non-O101/O162 O-serogroup *E. coli* isolates from our bacteremia collection (Fig. 2B) and similar to MDR rates recently described for the pandemic *E. coli* O25b-ST131 clone^29^. High levels of MDR among *E. coli* O101/O162 isolates were observed across most countries indicating that AMR is a specific trait of these strains rather than a general effect of increased resistance levels in certain countries. Furthermore, *E. coli* O101/O162 strains displayed relatively high resistance levels across all classes of clinically relevant antimicrobial drugs (Fig. 2C). Resistance rates to fluoroquinolones were particularly high (82.2–82.5%). Additionally, a considerable percentage (6.2%) of *E. coli* O101/O162 isolates was found to be resistant to last-resort carbapenem antibiotics.

### Methyltransferase gene disruptions are common in Onovel32 *rfb* loci of *E. coli* O101/O162 bacteremia isolates

To further classify O101/O162 O-serogroup *E. coli* isolates, we performed an in-depth sequence analysis of the *rfb* loci of these isolates. We observed that the Onovel32 *rfb* loci showed little sequence variation overall. However, for a subset of isolates (58/191, 30.4%) the putative MT encoding gene was found to be disrupted (these *rfb* loci are hereafter referred to as Onovel32MT-). For some strains this was due to small deletions leading to a frameshift and premature stop codon in the protein, in other cases (17/58) the disruption appeared to be the result of an insertion of a transposable element within the MT encoding gene as detected by a BLAST search of the gene against a reference database of insertion element (IS) sequences^30^ (Supplementary Fig. S1, Supplementary Data S1). The insertion location and type of IS element varied per strain, although in most cases IS1 was detected. The variety of different mutations point towards a selective pressure against expression of the MT or a beneficial effect of a functional inactivation or alteration.

### *E. coli* O101/O162 bacteremia isolates branch into several dominant lineages

**Fig. 3:**
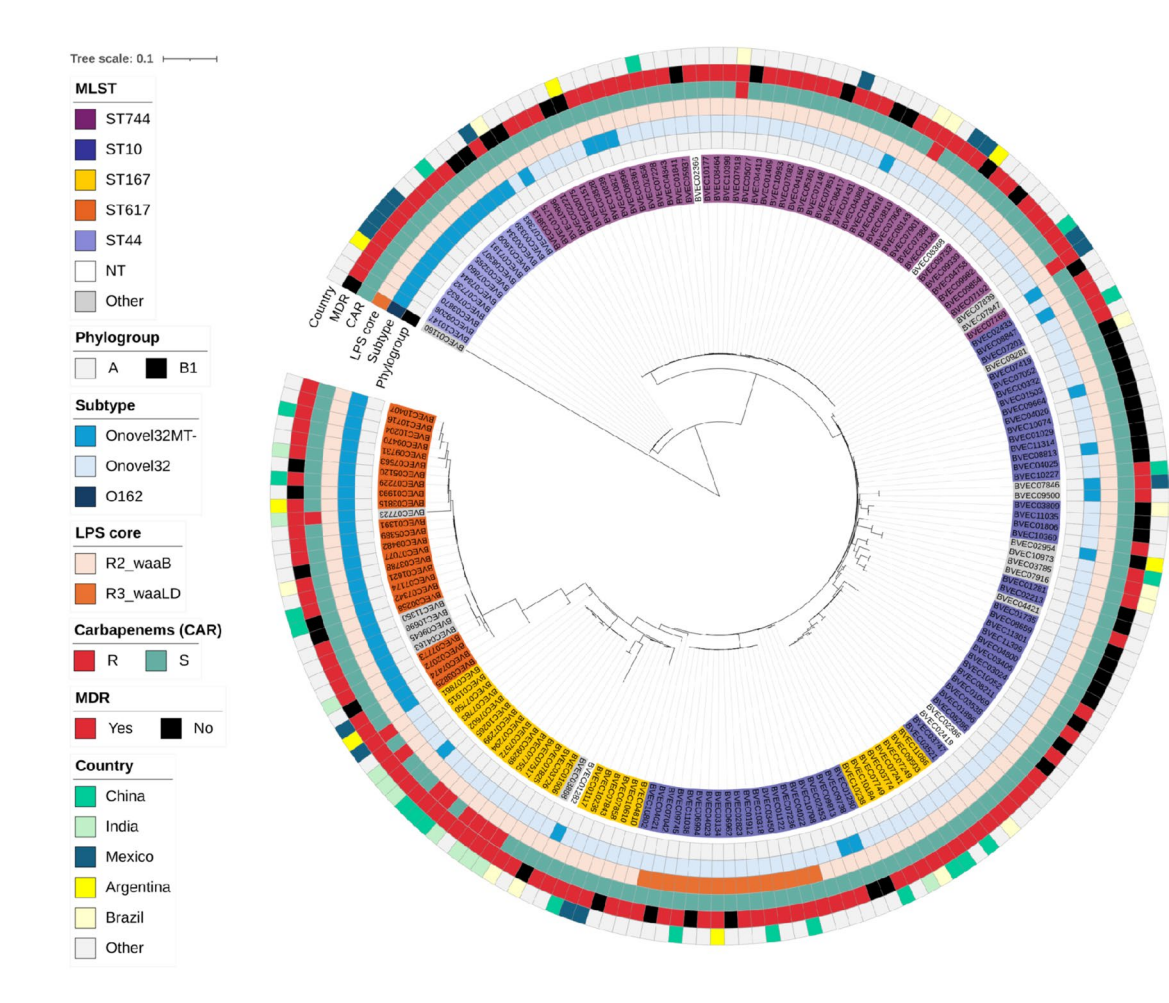
Midpoint rooted phylogenetic tree representing the phylogeny of *n* = 192 *E. coli* bacteremia isolates containing O101, O162 or Onovel32 *rfb* loci. Isolate IDs are colored according to sequence types (MLST), of which the five most prevalent are indicated. Colored rings (inside to outside) indicate (1) phylogroup, (2) identified *rfb* (sub)types, (3) LPS core type (R2 or R3, identified by *waaB* or *waaL* and *waaD* gene sequences, respectively), (4) carbapenem resistance (S = sensitive, R = resistant), (5) MDR and (6) country of origin, where countries with > 5% O101/O162 O-serogroup prevalence are indicated.

We next performed whole genome sequence (WGS)-based analyses including Clermont phylotyping and multi-locus sequence typing (MLST) to further delineate the full set of *n* = 192 O101/O162 O-serogroup ExPEC isolates. A WGS-based phylogenetic reconstruction was built to examine strain relatedness (Fig. 3). All isolates were classified as phylogroup A with the exception of a single isolate containing an O162 *rfb* locus, which was classified as B1. The isolates mainly clustered based on sequence type, of which five were dominant, being ST10 (*n* = 57, 29.7%), ST744 (*n* = 47, 24.5%) ST167 (*n* = 29, 15.1%), ST617 (*n* = 23, 12.0%) and ST44 (*n* = 14, 7.3%), all part of the widely disseminated clonal complex 10 (CC10)^31^. Of note, in our full analysis set of 7439 *E. coli* bacteremia isolates, sequence types ST167, ST44, ST617 and ST744 were exclusively associated with the O101/O162 O-serogroup and not with any other O-serotype. Remarkably, all ST617 and ST44 isolates were found to carry an Onovel32MT- *rfb* locus, wherein specific MT gene disruptions were identical within these STs suggesting clonal spread. Isolates belonging to these lineages displayed a relatively large phylogenetic distance towards ST744, ST10 and ST167 isolates, that clustered relatively closely to each other in the tree. The Onovel32MT- subtype was observed among other sequence types in a minority of isolates only. MDR was found across isolates within all lineages, with a particularly high prevalence among ST167 (28/29, 96.6%). Notably, most (8/12) of the carbapenem resistant isolates were found to cluster within ST167 and contained an *bla*NDM-5 carbapenemase gene (Supplementary Data S1), an association which has been described before^18^.

### ExPEC strains of O101/O162 O-serogroup subtypes express different LPS profiles

**Fig. 4:**
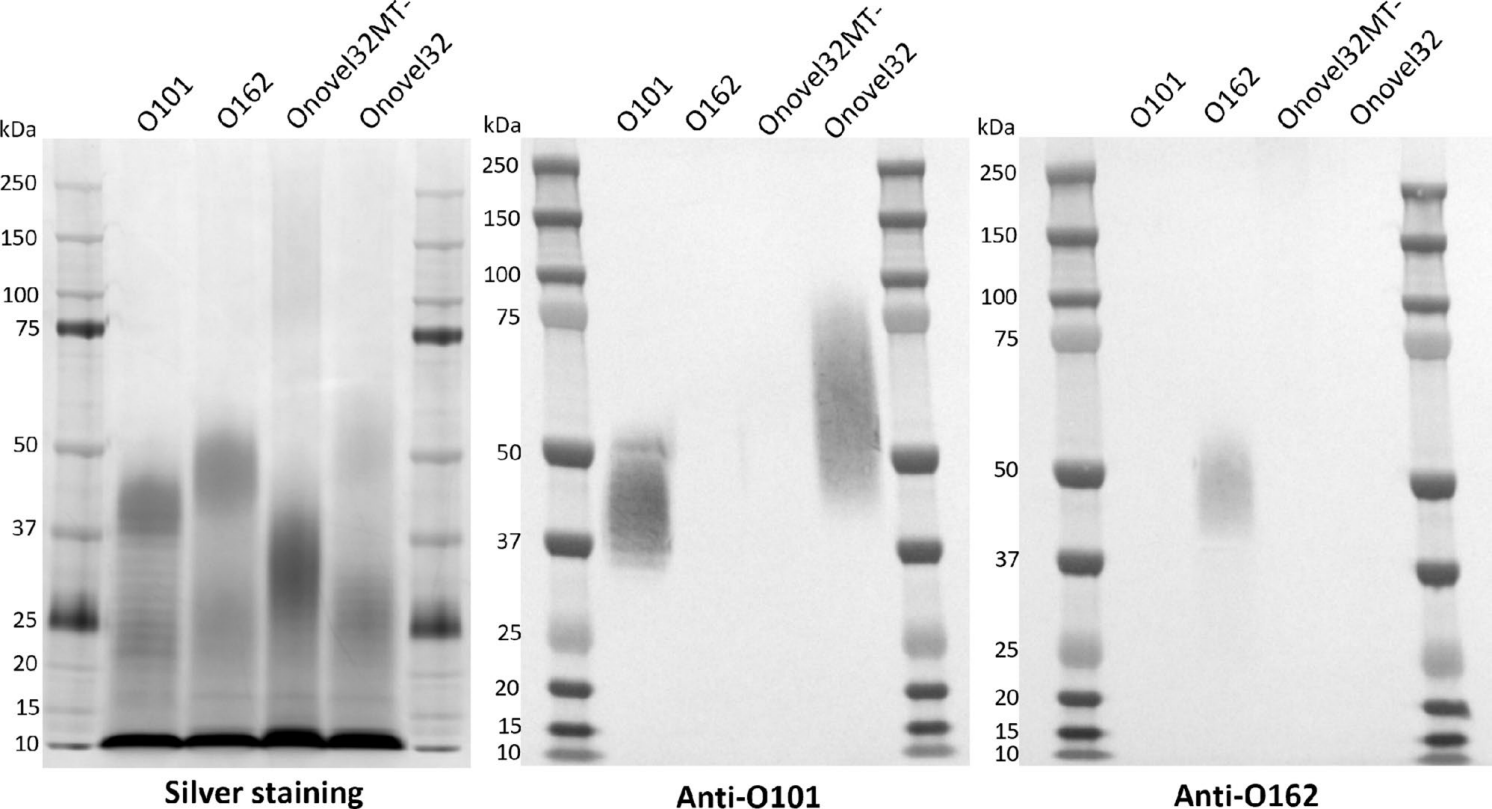
O-antigen profile of *E. coli* O101/O162 strains. Representative profiles of LPS extracted from *E. coli* strains containing *rfb* loci of O101, O162, Onovel32MT- and Onovel32 were visualized on silver stained SDS-PAGE and Western blot using *E. coli* O101 typing serum (SSI cat# 85095) or *E. coli* O162 typing serum (SSI cat #85158). Images were digitally cropped to align blots and improve clarity. Original gel and blot images are presented in Supplementary Fig. S2.

The genetic characterization of O101/O162 O-serogroup ExPEC strains resulted in the identification of two major O-serotype variants containing Onovel32 and Onovel32MT- *rfb* loci, that showed varying results in the agglutination assay using O-antigen specific antiserum. To further evaluate differences in O-antigen expression patterns, we characterized the LPS profile and typing serum reactivity for a subset of clinical isolates carrying O162, Onovel32 or Onovel32MT- *rfb* loci and an *E. coli* O101 reference strain (H510a, O101:K-:H33). We observed that most *E. coli* O101/O162 isolates showed a bimodal O-antigen chain length distribution with populations of relatively short (~ 15–35 kDa) and long (~ 35–100 kDa) chain lengths (Fig. 4, Supplementary Fig. S2, Supplementary Fig. S3). This is unusual for *E. coli*, as other serotypes generally show a unimodal O-antigen chain length distribution. Only the population of long chain O-antigen from the O101 reference strain and from clinical isolates containing intact Onovel32 *rfb* loci was detected by O101 typing serum. This suggests structural differences between the populations of short and long chain O-antigen expressed by the same strain, with an apparent lack of O101 serum-specific epitopes on short chains. Similarly, only the population of long chain O-antigen from the O162 *rfb* containing clinical isolate was recognized by O162 typing serum. Additionally, these results show that ExPEC strains carrying an intact Onovel32 *rfb* locus express an O101 rather than O162 O-antigen, which is largely in line with agglutination results (Table 1). Despite similarities in LPS profile and antibody reactivity between the *E. coli* O101 reference strain and Onovel32 *rfb* containing clinical isolates, a broader O-antigen chain length distribution with longer maximum chain length and a relatively higher abundance of short chains was observed for the latter (Fig. 4, Supplementary Fig. S3). These differences may be linked to sequence variation between the O101 and Onovel32 *rfb* loci, particularly within the GT- and MT-encoding genes (Fig. 1). Interestingly, the LPS profile of *E. coli* isolates containing Onovel32MT- *rfb* loci was markedly different from those with intact *rfb* loci. For the Onovel32MT- *rfb* containing isolates, we observed a unimodal O-antigen chain length distribution and complete absence of O101 serum reactivity, indicating a major effect of MT disruption on both O-antigen structure and chain length. None of the tested Onovel32MT- *rfb* containing strains were recognized by O162 typing serum on Western blot (Supplementary Fig. S3). This contrasts with results from the agglutination assay where we identified seven Onovel32 *rfb* containing *E. coli* isolates agglutination positive for O162, four of which contained disruptions in the MT encoding gene (Table 1). Interestingly, two of the other three O162 agglutination positive isolates showed sequence variation in the otherwise highly conserved glycosyltransferse directly downstream of the MT encoding gene (designated GT4). The LPS profile was determined for one of these two strains, showing a similar pattern as Onovel32MT- containing *E. coli* isolates (Supplementary Fig. S3). These results suggest that disruptions of the MT and sequence changes in GT4 can affect the O101 polysaccharide structure resulting in altered reactivity to O-antigen specific antiserum.

Altogether, our results point towards structural heterogeneity of the O-antigens expressed by *E. coli* O101/O162 strains and reveal that the two dominant subtypes identified among human bacteremia isolates, containing Onovel32 and Onovel32MT- *rfb* loci, are O101 variants that are serologically different from each other and deviate from the classical O101 and O162 structures.

### O-polysaccharides from the two dominant variants within O101/O162 O-serogroup ExPEC strains are structurally different

**Fig. 5:**
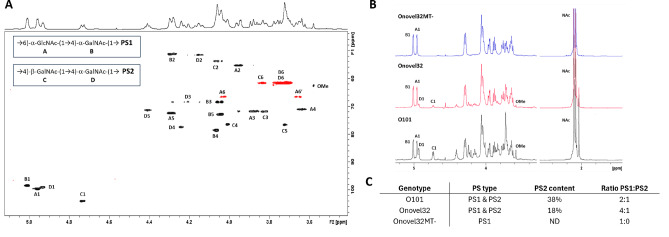
NMR spectra of O-PS samples recorded at 600 MHz and 50 °C. **(A)** An expansion of the HSQC-DEPT spectrum of O101 O-PS, methylene signals are inverted (in red). The PS1 and PS2 repeat unit proton/carbon crosspeaks have been labeled (A-D). **(B)** An overlay of the 1D DOSY spectra of the O101, Onovel32 and Onovel32MT- O-PS samples with diagnostic peaks labeled. **(C)** PS2 content determined from integration of the 1D ^1^H NMR spectra (B1 for PS1 and C1 for PS2).

To determine structural differences between O101-expressing *E. coli* strains at the O-antigen RU level, we analyzed O-polysaccharide (O-PS) extracted from LPS of the *E. coli* O101 reference strain and ExPEC clinical isolates carrying Onovel32 and Onovel32MT- *rfb* loci. 1D and 2D NMR experiments performed on O101 O-PS identified key diagnostic signals for PS1 (GlcNAc-GalNAc disaccharide) and PS2 (GalNAc disaccharide) as shown in the labeled HSQC spectrum (Fig. 5A); the assignments are in agreement with previous studies^22^. Analysis of the 1D NMR spectra indicated that both the O101 and Onovel32 O-PS samples predominantly consist of PS1, with PS2 present as a minor fraction (Fig. 5B and C). However, O101 showed a PS1:PS2 ratio of 2:1, in line with literature^22^, whereas this ratio was 4:1 for Onovel32. O-PS extracted from the Onovel32MT- strain was found to consist exclusively of PS1, as no PS2 signals could be detected. Additionally, a cross-peak attributable to an *O*-methyl group at 3.56/62.5 ppm (Fig. 5A) was present in the 1D NMR spectra of O101 and Onovel32 O-PS samples but not in Onovel32MT- O-PS (Fig. 5B), suggesting that the *O*-methyl residue is present on the PS2 RU. This is in line with our observation that the abundance of *O*-methyl appeared to be proportionate to the PS2-specific signals. Integration of the ^1^H NMR spectra gave a ratio of 0.18 and 0.12 methyl groups compared to C1 for O101 and Onovel32 O-PS, respectively (data not shown).

**Fig. 6:**
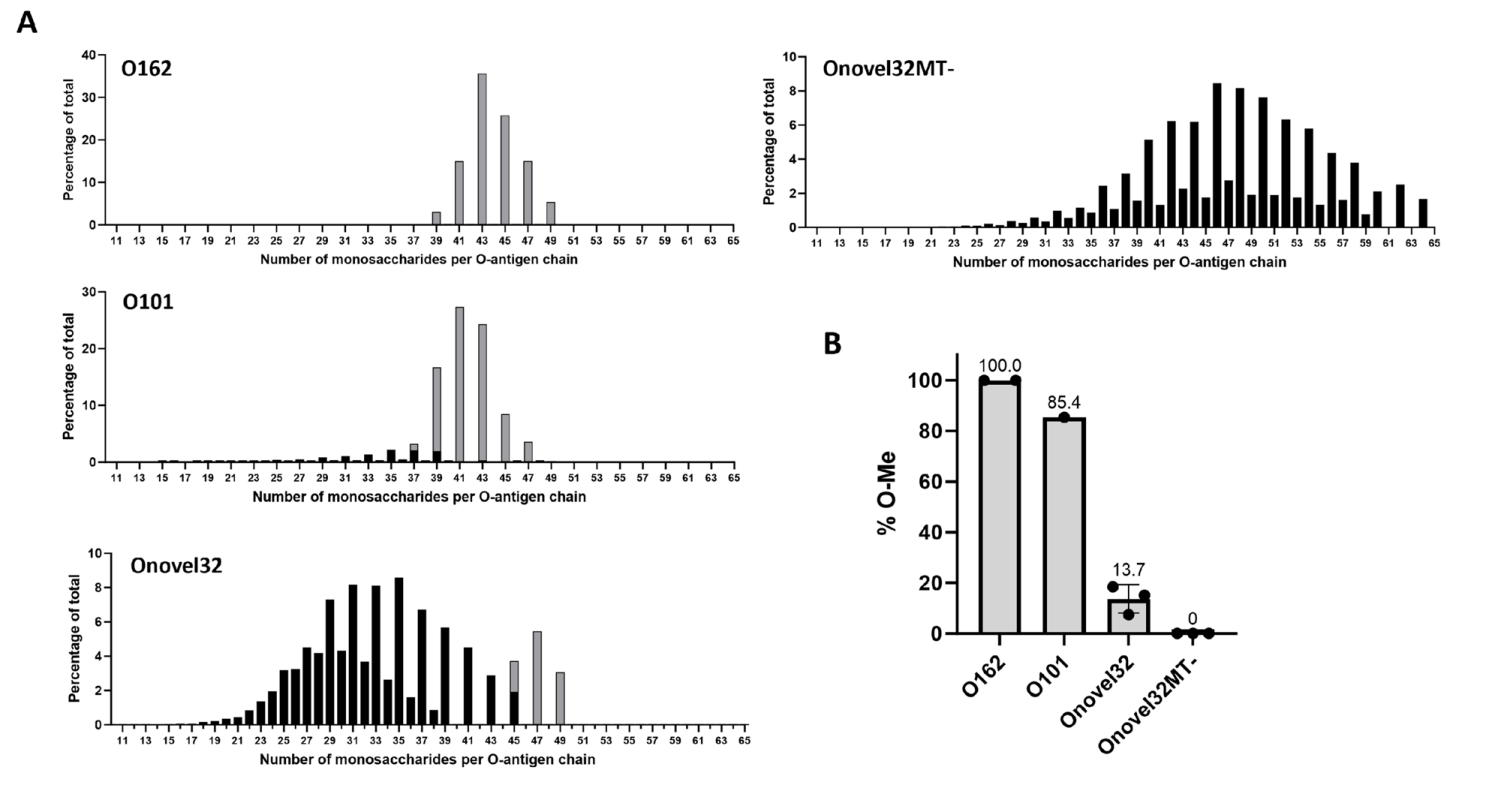
O-antigen chain length distribution and methylation of *E. coli* O101/O162 LLO. **(A)** Quantitative results derived from MALDI FT-ICR mass spectra showing the chain length distribution of polysaccharide structures from representative *E. coli* LLO samples from O162, O101, Onovel32 and Onovel32MT- *rfb* containing strains. Grey bars indicate polysaccharide chains on which a signal corresponding to one methylation was detected, black bars indicate non-methylated chains. **(B)** Average percentage of *O*-methylated polysaccharide chains determined for *E. coli* O101/O162 LLO samples. Circles represent independent measurements for individual *E. coli* strains representative for the subtypes.

To further characterize the O101/O162 O-PS, we analyzed lipid-linked oligosaccharides (LLOs) extracted from ExPEC isolates using high-resolution MALDI Fourier transform ion cyclotron resonance (FT-ICR) mass spectrometry^32^. LLOs are O-PS precursors which are located in the periplasm of *E. coli*. Based on the acquired mass spectra, three major types of polysaccharide distributions were observed for O101 and Onovel32 samples: (i) those with an even number of *N*-acetylhexosamine (HexNAc) monosaccharides, (ii) those with an uneven number of HexNAc monosaccharides and (iii) those with an uneven number of HexNAc monosaccharides and one methylation (Fig. 6A). Methylated monosaccharides were detected only on long polysaccharide chains with an uneven number of HexNAc monosaccharides and were absent from Onovel32MT- samples, in line with NMR results. The relative percentage of methylated polysaccharide chains was on average considerably higher for the O101 *E. coli* reference strain (85.4%) as compared to the average of three analyzed Onovel32 ExPEC clinical isolates (13.7%) (Fig. 6B). Of note, LLOs extracted from the two analyzed O162 *E. coli* strains were fully modified with a single methyl residue on each polysaccharide chain. In general, chain length distributions for LLO samples were in line with LPS profiles (Fig. 4), although for Onovel32MT- the number of monosaccharides per O-antigen chain was larger than expected. This might be attributable to technical factors, such as biases in extraction or detection methods, or could also be related to differences between LLO and LPS composition.

The results from our structural and biochemical analyses showed that O-antigen polysaccharides expressed by Onovel32MT- *rfb* containing ExPEC strains consist exclusively of PS1 RU and are not recognized by typing serum. This indicates that the epitopes that are recognized by the polyclonal antibodies present in O101 typing serum are likely located in the (methylated) PS2 RU. Moreover, the lack of reactivity to O101 typing serum of short chain polysaccharides present in O101 and Onovel32 *E. coli* strains implies that only the population of long polysaccharide chains present in these strains contains PS2. Additionally, these long chain polysaccharides contain single *O*-methyl residue modifications, suggesting that PS2 containing O101 O-antigens have nonreducing terminal *O*-methyl groups. The absence of PS2 and *O*-methyl modifications in polysaccharides of Onovel32MT- *rfb* containing ExPEC strains indicates that these structural features are dependent on an intact MT gene.

### Differences between opsonophagocytic properties of antibodies elicited by Onovel32 and Onovel32MT- polysaccharide conjugates

**Fig. 7:**
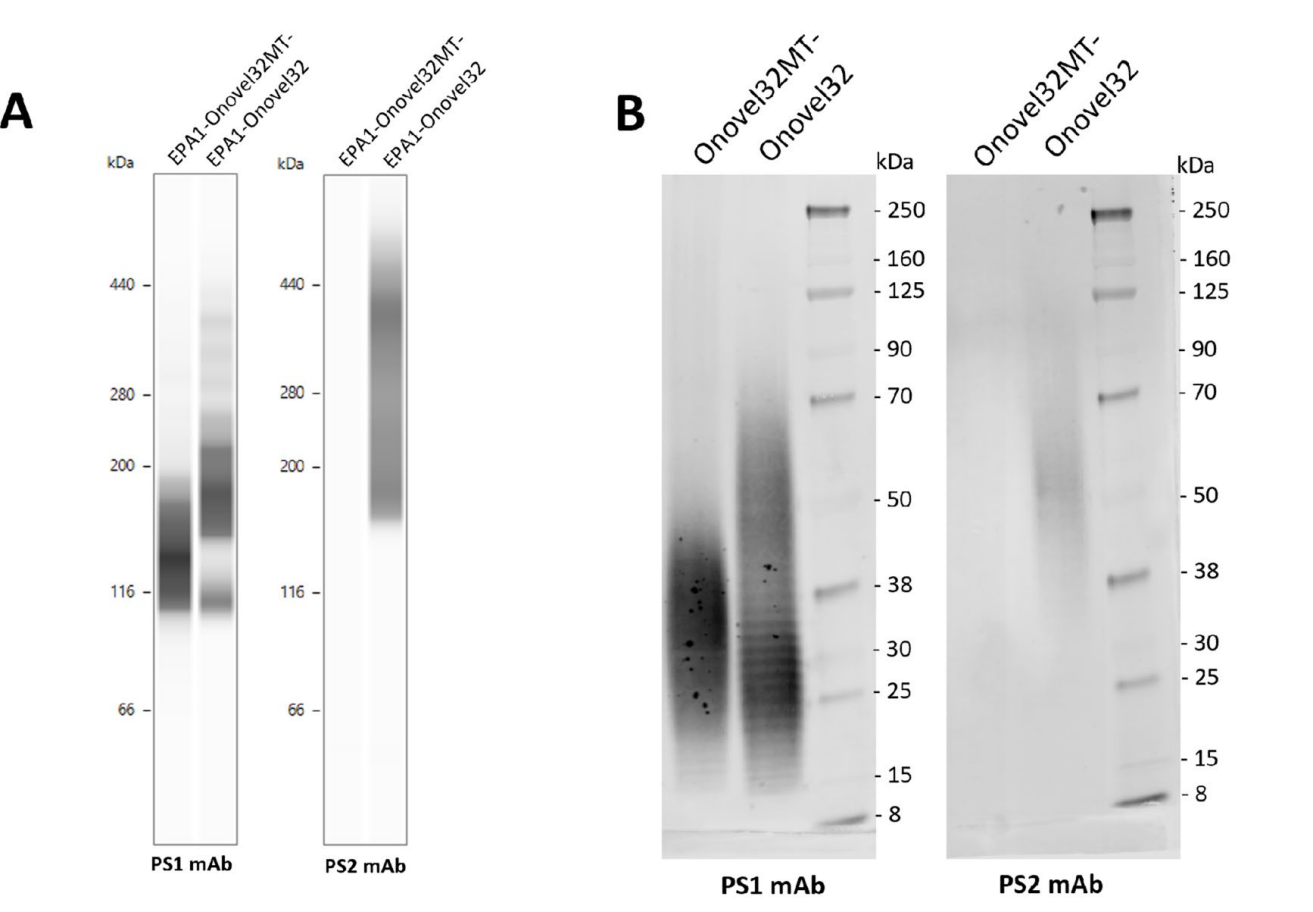
O-antigen profile of bioconjugates. **(A)** Image from capillary Western blot assay on EPA bioconjugates mono-glycosylated with Onovel32 or Onovel32MT- polysaccharides at a single glycosylation site (EPA1-Onovel32 and EPA1-Onovel32MT-, respectively), stained with anti-PS1 or anti-PS2 monoclonal antibodies. **(B)** Western blot on LPS samples extracted from representative *E. coli* strains containing *rfb* loci of Onovel32 and Onovel32MT-, stained with the same anti-PS1 and anti-PS2 monoclonal antibodies. Images in panel B were digitally cropped to align blots, display samples relevant for comparison with panel A and improve clarity. Original blot images are presented in Supplementary Fig. S2.

**Fig. 8:**
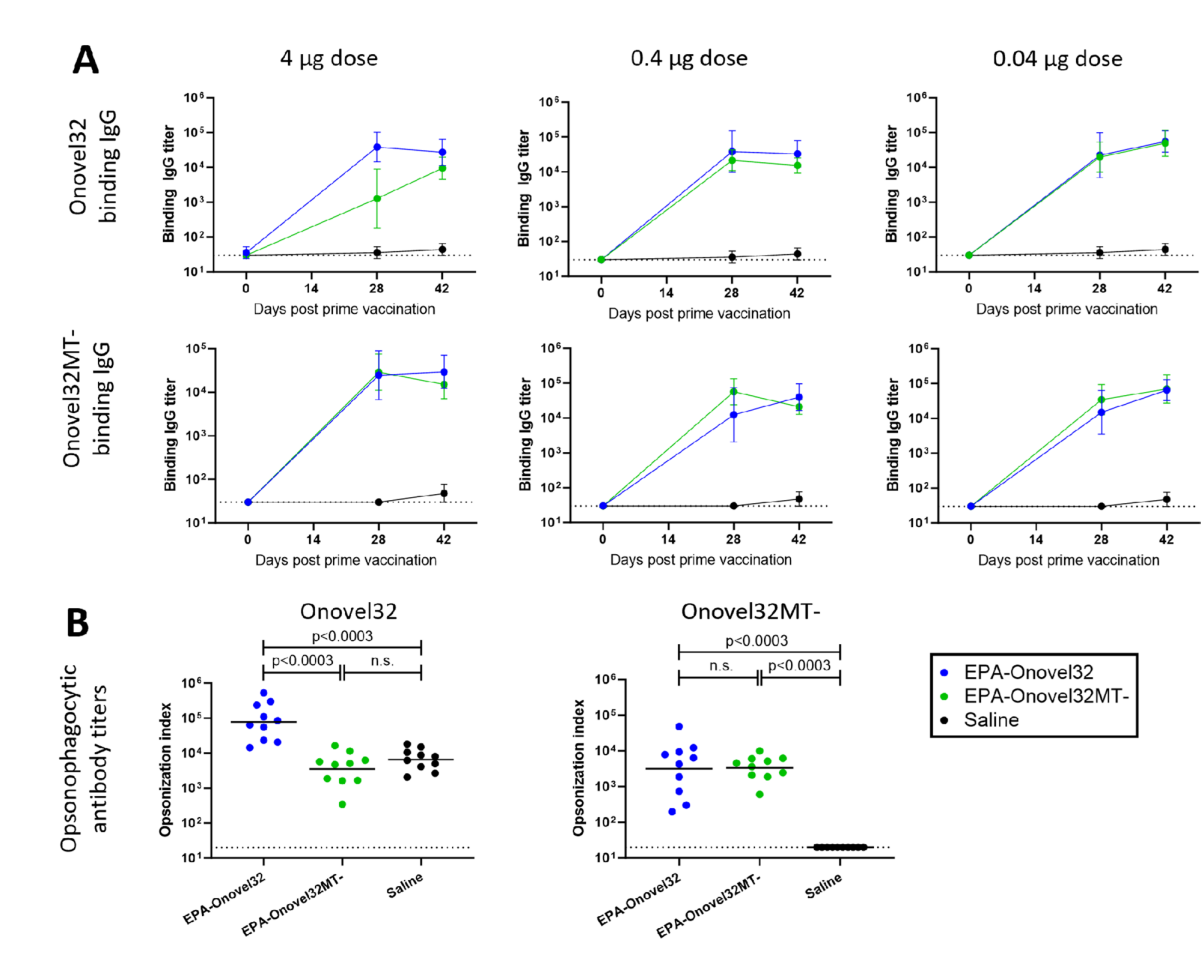
Sprague Dawley rats (*n* = 10 per group) were i.m. immunized with 4, 0.4–0.04 µg of EPA-Onovel32 (indicated in blue), EPA-Onovel32MT- (indicated in green) or saline (indicated in black) on days 0, 14 and 28. (**A**) Serum samples obtained at days 0, 28 and 42 were analyzed by ELISA for IgG antibodies binding to Onovel32 (upper panels), or Onovel32MT- (lower panels). Geometric mean EC50 titer +/- 95% confidence intervals are indicated, and the lower limit of detection is represented with a dotted line. (**B**) Serum samples obtained at day 42 from the animals immunized with 0.4 µg EPA-Onovel32 (indicated in blue), EPA-Onovel32MT- (indicated in green) or saline (indicated in black) were analyzed for opsonophagocytic antibodies against *E. coli* strain Onovel32 (left panel) or Onovel32MT- (right panel). Each circle represents the opsonization index of an individual animal, group geometric means are indicated with horizontal lines, and the LOD is indicated with a dotted line. Statistical analysis of OI titers was performed in GraphPad Prism (version 10.1.2) using the non-parametric Mann-Whitney test and a 3-fold Bonferroni correction for multiple testing.

Our studies led to the identification of two distinct bacteremia-associated O101-variants within the *E. coli* O101/O162 O-serogroup that showed structural differences at the polysaccharide level. We next investigated the potential of polysaccharide-conjugate constructs specific for these subtypes to elicit antibodies that could cross-react and facilitate opsonophagocytic killing of O101/O162 O-serogroup ExPEC strains. To this end, we produced bioconjugates containing Onovel32 or Onovel32MT- polysaccharides from *E. coli* strains genetically engineered to facilitate *N*-linked bioconjugation on four sites of an introduced carrier protein (genetically detoxified exotoxin A (EPA)). Using monoclonal antibodies specifically recognizing PS1 and PS2 structures, we could confirm on similarly produced mono-glycosylated bioconjugates that antibody reactivity and polysaccharide patterns were comparable to LPS extracted from ExPEC isolates, showing that bioconjugates are representative for the two O101 subtypes (Fig. 7, Supplementary Fig. S2). Antiserum obtained from rats immunized with multi-glycosylated EPA-Onovel32 and EPA-Onovel32MT- bioconjugates was then tested in an ELISA assay to determine titers of antibodies binding to purified LPS of Onovel32 and Onovel32MT- subtypes. Both conjugates were found to be highly immunogenic, as near- maximum titers were already obtained after two unadjuvanted immunizations with a 0.04 µg polysaccharide dose (Fig. 8A). No differences were observed between the two groups, as both EPA-Onovel32 and EPA-Onovel32MT- bioconjugates were found to elicit antibodies that cross-recognized LPS from both subtypes equally well. Subsequently, antibody functionality was assessed in an opsonophagocytic killing assay, wherein the animal serum from groups that received a 0.4 µg PS immunization dose was incubated with human complement and phagocytic cells to assess killing of Onovel32 and Onovel32MT- ExPEC target strains. Surprisingly, while antibodies induced by the EPA-Onovel32 bioconjugate facilitated killing of both Onovel32 and Onovel32MT- ExPEC target strains, immunization with EPA-Onovel32MT- mediated phagocytic killing of only the matching Onovel32MT- but not the Onovel32 ExPEC strain (Fig. 8B), despite the shared PS1 RU structure in both polysaccharides. These results show that the structural differences between polysaccharides of the two clinically relevant subtypes within the *E. coli* O101/O162 O-serogroup are an important determinant for a functional immune response against O101/O162 O-serogroup ExPEC strains.

## Discussion

In this study, we characterized a previously collected global set of O101/O162 O-serogroup bacteremia *E. coli* isolates^6,27,28^. *E. coli* O101/O162 was found to be globally widespread and highly associated with MDR. Infections with drug resistant *E. coli* pose significant challenges to treatment, and the increasing prevalence of high-risk clones is of major concern to public health. Notably, we observed that in countries for which high rates of AMR among *E. coli* have been described, including Mexico, China and India^33^, the proportion of the O101/O162 O-serogroup among bacteremia isolates was relatively high (> 6% versus 2.6% overall). Almost all *E. coli* O101/O162 isolates analyzed in this study were identified as belonging to phylogroup A with sequence types within CC10. These lineages are frequently observed among human extraintestinal infections^15,17^, but are also commonly associated with intestinal carriage and intestinal pathogenic *E. coli* (InPEC) pathotypes^31,34,35^. One of the lineages we observed to be associated with *E. coli* O101/O162 was ST167, which is of particular concern because of its resistance to last-resort carbapenem antibiotics^18,19,36–38^, a characteristic also found in the current study in addition to its association with MDR. Our results underline the notion of *E. coli* ST167 as a high-risk lineage^31^. We observed that *E. coli* O101/O162 isolates across all lineages showed increased levels of resistance to several classes of antimicrobial drugs that are often used for treatment of ExPEC infections, including fluoroquinolones for which over 80% of isolates were non-susceptible. Continued surveillance is important to effectively manage such infections and the development of preventive measures including vaccines will be crucial to limit the rise of infections caused by drug-resistant *E. coli* strains such as those belonging to the O101/O162 O-serogroup.

The results from our genetic and biochemical analyses showed that the *E. coli* O101/O162 bacteremia isolates in our collection almost exclusively contain an Onovel32 *rfb* gene cluster and that most of these isolates express an O-antigen that is structurally and serologically similar to O101. O-serotype O162 seems to be of much lower clinical relevance. In fact, our biochemical analysis indicated that Onovel32 *rfb* containing *E. coli* isolates with a positive agglutination response for O162, express O101 variant polysaccharides. Given the structural heterogeneity of O101 and O162 polysaccharides with shared PS1 RU^22^, it might be possible that the presence of a low level of PS1-specific antibodies in the O162 typing serum is responsible for the positive agglutination result of these strains.

**Fig. 9:**
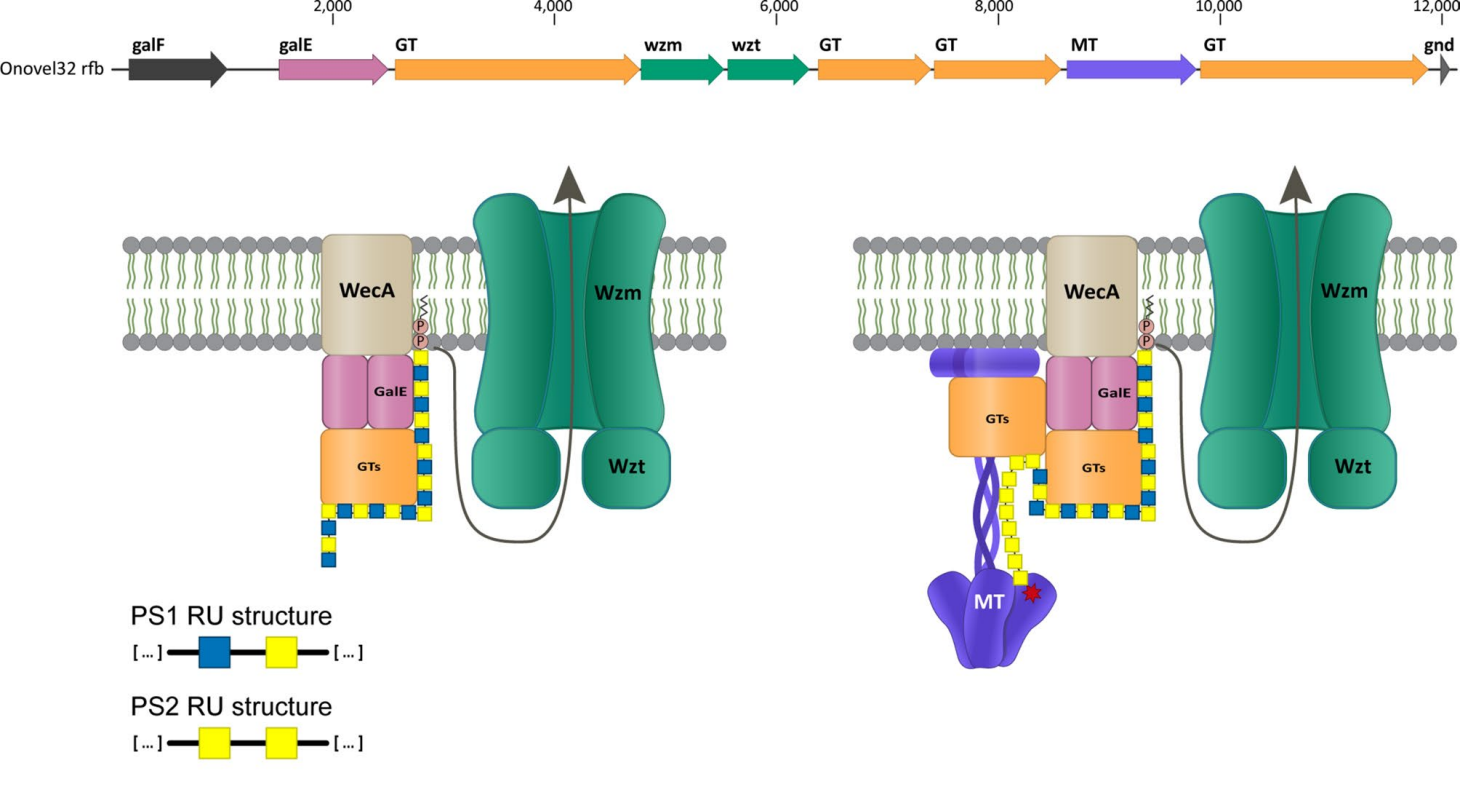
Proposed model for biosynthesis of *E. coli* O101 variant polysaccharides. O-antigen biosynthesis proteins encoded in the Onovel32 *rfb* locus (top) localize at the inner membrane to assemble and export the polysaccharide structure consisting of PS1 in absence of a functional MT-GT complex (left). Presence of an MT-GT complex may lead to an extension of the PS1 core polysaccharide with PS2 repeat units, which is methylated when the polysaccharide chain reaches the MTase domain (right). The monosaccharides are indicated with yellow (GalNAc) and blue (GlcNAc) squares, the methyl residue is indicated with a red star. *galE* (pink) encodes an UDP-glucose-4-epimerase involved in the conversion of UDP-GlcNAc to UDP-GalNAc. GTs (orange) encode glycosyltransferase enzymes responsible for the linkage between the individual monosaccharides within the polysaccharide chain, *wzm* and *wzt* (green) encode the ABC transporter responsible for translocation of the polysaccharide to the periplasm, MT encodes the methyltransferase enzyme responsible for the addition of a terminal *O*-methyl residue on the polysaccharide chain. WecA is an integral membrane protein encoded outside the *rfb* locus, that functions as an undecaprenyl phosphate GlcNAc-1-phosphate transferase and initiates O-antigen biosynthesis.

In-depth *rfb* locus sequence analysis and phenotypic LPS profiling revealed that two major variants exist among the Onovel32 *rfb* containing ExPEC isolates in our collection. This is the result of disruptive mutations or IS elements in the *rfb* encoded MT in 30% of strains, with functional consequences on O-antigen biosynthesis. Structural studies showed that the Onovel32MT- genotype translates into an alteration of polysaccharide chain length distribution and composition, with a complete loss of PS2 RU as opposed to the mixture of PS1 and PS2 RU that is present in O-polysaccharides of *E. coli* strains that contain an intact Onovel32 *rfb* locus and of the O101 reference strain. We also showed that presence of an intact MT encoding gene is a prerequisite for O-polysaccharide chain modification with an *O*-methyl residue, indicative for end-capping, which has not been previously experimentally shown for *E. coli* O101/O162. Together, the results from our biochemical and structural analyses suggest a model where the O-PS of O101 and Onovel32 *rfb* containing *E. coli* strains is heterogenic and consists of populations of short-chain polysaccharides consisting of PS1 RUs only and of long chain polysaccharides that are hybrid structures with a core consisting of PS1 RUs that are elongated by PS2 RUs at the non-reducing end and can be end-capped with an *O*-methyl residue. This model is based on the following observations: (i) O-PS from the O101 reference strain shows a higher relative abundance of PS1 as compared to PS2 (Fig. 5), (ii) shows a higher abundance of long-chain versus short chain polysaccharides (Figs. 4 and 6), and (iii) PS2-specific antiserum reacts to long chain polysaccharides only, while PS1-specific antiserum reacts to the full spectrum of chain lengths (Figs. 4 and 7). This model probably also applies to O162, for which RU structures are modified with an additional ara4dHex side branch^22^. Although unique for *E. coli*, similar hybrid structures have been described for *K. pneumoniae* serotypes O1 and O2ac, where respectively O1 or O2c RU are attached to the non-reducing terminus of an O2a disaccharide RU core structure^39^. The genetic determinants that link these RUs for *K. pneumoniae* O1 and O2ac have been identified. However, the factor that could potentially attach PS2 to the non-reducing terminus of the PS1 RU for *E. coli* O101/O162 O-antigens is currently unknown. Possibly, one of the four glycosyltransferase enzymes encoded in the *rfb* locus is responsible. Two of these enzymes (the first and the last in the *rfb* locus, designated *werR* and *werT*in Liu et al.^5^.) appear to contain two catalytic sites, suggesting potential bi-functional activity. Interestingly, we found that disruption of the MT led to a complete loss of PS2 RUs and this enzyme therefore seems to be important for synthesis of the complete polysaccharide structure. However, as the MT contains one predicted methyltransferase (MTase) domain in the N-terminal part of the protein and a coiled coil structure at its C-terminus, it probably does not play a direct role in linkage of PS1 and PS2. The MT enzyme might work as a molecular ruler, similar to WbdD of *E. coli* O9a, where chain length termination occurs when the growing chain of polymannan RUs (polymerized by the glycosyltransferase enzymes) reaches the MTase domain located at the end of the coiled coil, which adds a methyl phosphate to the glycan at the nonreducing end of the polysaccharide chain^24,40^. For *E. coli* O101/O162, the MT may form a complex with one or more glycosyltransferase enzymes at the inner membrane, one of which could be responsible for the addition of PS2 to the backbone structure of PS1 RUs (Fig. 9). This could explain the loss of PS2 from O101 polysaccharides when the MT is disrupted, as such complex may no longer be formed. Interestingly, we observed in our collection of clinical ExPEC isolates one strain with an intact MT that showed the same LPS profile as MT mutant strains (Supplementary Fig. S3). This strain was found to contain a mutation leading to a change of one amino acid at an otherwise 100% conserved position in the predicted glycosyltransferase domain of the GT4 protein encoded directly downstream of the MT (encoded by *werT*according to Liu et al^5^.). No other *rfb* sequence differences were observed relative to the majority of Onovel32 *rfb* containing strains. Similarly, a previous report that described plasmid-based expression of O101 O-antigen in *E. coli* K12 showed that inactivation of the same glycosyltransferase enzyme had a comparable effect on O-antigen chain length distribution as we observed for Onovel32MT- *rfb* containing isolates^41^. These results suggest that the final glycosyltransferase enzyme in the *rfb* locus might work in concert with the MT to assemble complete PS2 containing O101 polysaccharide chains. In this model, sequence differences in the MT and GT encoding genes between classical O101 and Onovel32 *rfb* containing *E. coli* strains may affect enzyme activity and complex formation, resulting in the LPS profile differences we observed between these two variants. Novel studies that apply site-directed mutations and in vitro studies on processivity in enzymatic catalysis may shed more light on the biosynthesis mechanisms of O101 polysaccharide chains and the specificity of the four glycosyltransferase enzymes encoded in the *rfb* locus.

In addition to causing human extraintestinal infections as reported in this study and by others^15,42^ Onovel32 *rfb*-containing *E. coli* strains are also frequently isolated from infected animals^14,16,26,29^ and are present among diverse pathotypes. These strains may have a mix of resistance genes and virulence factors that could be beneficial for their survival in different environmental niches. Possibly, the mucoviscous phenotype that appears to be a hallmark for these strains^21^, which we also observed in the current study (Supplementary Fig. S4), may provide protective properties allowing the bacteria to survive and thrive under diverse and harsh conditions.

Structural variation of the O-antigen is an effective bacterial strategy to avoid immune recognition and clearance by the host’s immune system, and to avoid attack by bacteriophages that use the O-antigen as a receptor. The MT disruption in the Onovel32 *rfb* locus of *E. coli* O101/162 results in loss of the (methylated) PS2 RU, that appears to contain immunodominant epitopes (Fig. 4). The O-antigen structure expressed by our collection of contemporary Onovel32 *rfb* containing ExPEC strains contains a lower amount of PS2 as compared to the classical *E. coli* O101 reference strain H510a, that was isolated several decades ago. There may be an evolutionary benefit of the altered O-polysaccharide structure and chain length distribution resulting from reduction or loss of the immunogenic (methylated) PS2 RU. Interestingly, we observed that antibodies elicited by immunization with EPA-Onovel32 and EPA-Onovel32MT- polysaccharide conjugates had different functional properties. Antibodies induced by both immunogens could cross-recognize LPS from all O101/O162 variants, as expected given that the PS1 repeat unit is shared across all subtypes. However, the PS1-specific antibodies elicited by the EPA-Onovel32MT- conjugate could not facilitate opsonophagocytic killing of an *E. coli* Onovel32 strain above background levels, in contrast to the antibodies elicited by the EPA-Onovel32 conjugate, that include those specific for PS2. Since we observed that approximately 70% of *E. coli* O101/O162 bacteremia isolates contain intact Onovel32 *rfb* loci, these results suggest that the majority of ExPEC strains in this O-serogroup may potentially not be targeted for opsonophagocytic killing by PS1-specific antibodies. One possible explanation that would fit with our model of a copolymeric polysaccharide structure with a base of PS1 and extension of PS2, could be that the terminal PS2 RU might restrict access of antibodies to the underlying layer of PS1 on whole *E. coli* cells. However, preliminary results from whole-cell ELISA experiments where *E. coli* expresses its O-antigen in a more native form relative to ELISA with purified LPS, do not support this theory (Supplementary Fig. S5), although the in vitro assay conditions may not be representative for an infection in vivo. It remains to be determined which epitopes on the O101 polysaccharide structure are bound by functional antibodies. Such insights could increase our understanding of opsonophagocytic antibody activity and immune escape mechanisms by the pathogen which may be useful for future vaccine development efforts targeting *E. coli* O101/O162 strains.

Altogether, the results from our experiments have given important insights into the molecular epidemiology of *E. coli* O101/O162 associated with human bacteremia. We shed light on structural O-antigen variation within this O-serogroup which could be linked to the presence or absence of an intact MT encoding gene within the *rfb* locus. Our data supports a model for a hybrid polysaccharide structure, which is unique for *E. coli*. Furthermore, we have found that O-antigen variation within *E. coli* O101/O162 has important immunological consequences. The results from our studies underline the versatility of *E. coli* in the ways it can adapt to the host’s immune system and different environmental conditions, and stress the importance of monitoring the emergence of high-risk clones that can potentially outmaneuver our options to combat infections with this pathogen.

## Methods

### ExPEC strain collection

The collection of *E. coli* bloodstream isolates analysed in this study were sourced from previously described observational surveillance studies^6,27,28^. The analysis set includes 7439 *E. coli* isolates previously characterized for agglutination-based O-serotype and antibiotic resistance based on MIC values. These isolates were originally derived from adult bacteremia patients hospitalized in different countries across the world between 2011 and 2023^6,27,28^. MDR was defined as resistance to ≥ 3 classes of antimicrobial drugs as previously described^43^. Isolate-specific metadata and typing data is available in Supplementary Data S1.

### Genome sequencing and bioinformatic analysis

Genomic DNA was isolated from biomass of ExPEC strains using silica filter-based methods, after which libraries were prepared with Nextera XT (Illumina). DNA was subjected to whole genome sequencing using the Illumina NovaSeq 6000 or MiSeq systems generating paired-end sequence reads. Whole genome assemblies were generated using CLC Genomics workbench (Qiagen). The quality of all sequences was checked with CheckM (v1.1.3)^44^ and only genomes with a contamination threshold of < 5% and completeness threshold of > 95% were included in the analysis. Multi-Locus Sequence Typing (MLST) was performed by mlst (https://github.com/tseemann/mlst), using the Achtman scheme for *E. coli*. *E. coli* phylogroups were defined according to the ClermonTyping method^45^. Genomes were annotated using Prokka^46^. In silico serotyping was performed using Kaptive^47^ and Abricate (https://github.com/tseemann/abricate) with BLAST+ (NCBI) using a custom database including genbank files and *wzm*/*wzt* genes of the *rfb* loci of Onovel32 (SRR2544773), O101 (AB812046), O162 (AB812067) and O89 (AB812038). Full *rfb* gene loci were compared using Clinker^48^ and CLC Genomics Workbench (Qiagen).

The Onovel32MT- genotype was assigned by detecting the MT encoding gene (annotated as *ubiG2*) using Abricate v1.0.1 with a 20% coverage and 80% identity cutoff followed by manual inspection of the column “COVERAGE_MAP” of every hit for the presence of truncations or frameshifts within the gene or the distribution of the gene on 2 different contigs. MT encoding gene sequences and up to 2000 nucleotide up- and downstream flanking regions were extracted and queried against the ISfinder repository^30^, using BLASTN v2.16.0+. BLAST output was manually inspected to identify evidence for IS elements within the MT encoding genes. For phylogeny, Parsnp v1.7.4^49^ was used to align *E. coli* genomes and identify single nucleotide polymorphisms (SNPs). The SNP-based phylogenetic tree was constructed using RAxML 8.2.12^50^ with the maximum likelihood method. The tree was visualized along with meta-data in iTOL v7^51^ using a midpoint rooted tree.

### Determination of LPS profile

To obtain LPS samples from *E. coli* strains, biomass was collected from cultures grown overnight on LB agar plates at 37˚C. Biomass was normalized to an OD_600_ of 1.0 in LDS sample buffer with reducing agent, boiled for 10 min at 95˚C and thereafter treated with 400 µg/ml Proteinase K (Invitrogen) for 1.5 h at 60˚C. An equivalent of 0.1 OD_600_ was loaded onto 4–12% Bis-Tris NuPage gels (Thermo Fisher Scientific), which were run for 40 min at 200 V before being subjected to either silver staining or transferred to nitrocellulose for Western blot. Silver staining was performed by the acidic method^52^. Western blot was performed using 1:50 diluted monospecific rabbit antiserum from SSI diagnostica (cat# 85095 (O101), cat #85158 (O162)) or PS1 and PS2 specific monoclonal antibodies obtained as described below. Capillary Western blot was performed on periplasmic extracts from glycoconjugate production strains at 25 µg/ml protein concentration with 1:50 diluted antibodies using JESS (ProteinSimple) and standard manufacturer’s instructions.

### NMR spectroscopy

LPS was purified from biomass of O101, Onovel32 and Onovel32MT- *E. coli* strains using a phenol-free method, followed by mild hydrolysis to obtain O-PS (LPS Biosciences, Orsay, France). O-PS samples (3–5 mg) were dissolved in deuterium oxide (D_2_O, 99.9%, Sigma-Aldrich) and subjected to two cycles of D_2_O exchange before transfer to a 5 mm tube (Bruker). 1D (^1^H and DOSY) and 2D, DOSY-TOCSY and HSQC-DEPT NMR spectra were obtained using a Bruker Avance III 600 MHz NMR spectrometer equipped with a BBO Prodigy cryoprobe. The probe temperature was set at 50 °C and the spectra recorded and processed using standard Bruker software (Topspin 3.6.2). The 1D proton spectra were recorded using a 30 degree pulse and a D1 of 2 s; and used for integration. The 1D DOSY (ledbpgp2s1d) experiment was optimized to remove signals from low molecular weight compounds such as solvent. The 2D TOCSY experiment (ledbpgpml2s2d) was performed using mixing time of 180 mS and the ^1^H-^13^C HSQC-DEPT experiment (hsqcedetgpsisp2.3) was optimized for J = 145 Hz. 2D experiments were recorded using non-uniform sampling: 40% for homonuclear and 20% for heteronuclear experiments. Spectra were referenced relative to H1/C1 of H1/C1 of 4)-α-D-GalpNAc (B) of PS1: ^1^H at 5.01 ppm, ^13^C at 98.4 ppm^22^.

### MALDI FT-ICR mass spectrometry

MALDI FT-ICR mass spectrometric analysis of LLO was performed as previously reported, with minor modifications^32^. LLO were extracted from *E. coli* lyophilized biomass with a 10:10:3 (v/v/v) chloroform: methanol: water (CMW) solution and purified with a tC18-SepPak SPE cartridge (500 mg/6 ml, Waters). A second purification step was performed using C4-SPE tips (C4 ZipTip™, 0.6 µl resin, Millipore) to further improve the sensitivity and specificity of the analysis. For this step, 3 µl of the LLO extract were diluted with 3 µl of water and loaded onto a preconditioned C4-SPE tip. The tip was then washed three times with 15 µL of a 50:50 (v/v) methanol: water solution, and the LLO were eluted with 1.5 µl of an 80:20 (v/v) methanol:1mM NaCl solution. The elutes were directly spotted onto an AnchorChip MALDI target plate (Bruker Daltonics) along with 1 µl of 2,5-dihydroxyacetophenone (DHAP) solution. MS measurements were performed on a 15 T solariX XR FT-ICR mass spectrometer (Bruker Daltonics, Bremen, Germany) equipped with a CombiSource and a ParaCell (2xR). The MS system was operated in 1 omega mode using ftmsControl software (Bruker Daltonics). Data were acquired in positive ionization mode, and mass spectra were visually inspected using DataAnalysis v5.0 SR1 (Bruker Daltonics). Theoretical *m/z* values of intact LLO were generated in GlycoWorkbench v2.1 stable build 146^53^ and Windows Excel 2002.

### Production of bioconjugates

Production strains for bioconjugates were generated by genetic modification of *E. coli* W3110 as described previously^54^. Donor DNA of *rfb* gene clusters of Onovel32 and Onovel32MT- was PCR amplified from ExPEC isolates representative for the subtypes and used for genetic integration in the target strain by homologous recombination. Bioconjugates were produced by arabinose-inducible expression of *Pseudomonas aeruginosa* EPA or *Campylobacter jejuni* AcrA carrier proteins containing one or more motifs for *N*-linked glycosylation from an introduced plasmid and IPTG-inducible expression of *Campylobacter jejuni* oligosaccharyltransferase PglB from a second introduced plasmid. The produced bioconjugates were purified from periplasmic extracts by osmotic shock and multistep chromatographic purification as described^9^. Bioconjugates on AcrA and EPA proteins that contained one glycosylation motif and a C-terminal 6-HIS-tag were purified using Ni-NTA affinity chromatography.

### Animal immunization and generation of monoclonal antibodies

Eight weeks old female Sprague Dawley rats were immunized intramuscularly with 0.04, 0.4–4 µg of non-adjuvanted EPA-Onovel32 or EPA-Onovel32MT- bioconjugate (100 µl/dose) or saline at days 0, 14 and 28. Blood was collected one day prior to each immunization and at a terminal bleed on day 42. Female New Zealand White rabbits were immunized with 2 µg of EPA-Onovel32 or EPA-Onovel32MT- bioconjugate and CFA/IFA 1:1 (500 µl/dose) at days 0, 7, 10 and 18. Blood was collected at a terminal bleed on day 28. Monoclonal antibodies specific for O101 PS1 were obtained by hybridoma technology after immunization of rats with AcrA bioconjugates containing Onovel32 polysaccharides, followed by ELISA with Onovel32 and Onovel32MT- LPS as positive selection, and with O73-AcrA bioconjugate and BSA-Biotin as negative selection (ImmunoPrecise antibodies). Monoclonal antibodies specific for O101 PS2 were obtained by hybridoma technology after immunization of rats with EPA bioconjugate containing Onovel32 polysaccharides followed by ELISA with EPA-Onovel32 bioconjugate as positive selection, and with EPA-Onovel32MT- bioconjugate and EPA protein as negative selection (GenScript). The monoclonal VH/VL regions of selected clones were sequenced and recombinantly expressed, followed by IgG purification.

### ELISA

Onovel32 and Onovel32MT- specific IgG serum antibody titers were evaluated by ELISA on day 0, 28, and 42 serum samples in 384-well plate format, where plates were coated with 30 µl per well of 2.5 µg/ml purified LPS obtained by single-phase extraction from representative ExPEC isolates (LPS Biosciences) and 5 µg/ml methylated bovine serum in PBS. Plates were blocked with diluted skimmed milk in PBS with 0.05% Tween 20. Serum samples were added in a 12-step 3-fold serial dilution starting from 1:90. HRP-labeled goat-anti-rat secondary antibody (Biolegend, cat# 405405) was used at a 1:2000 dilution. Plates were developed using TMB substrate. After adding 1M H_3_PO_4_ stop solution, plates were read at 450 nm in a plate reader. Analysis was performed using Gen5 software v3.04 (Agilent). Optical density (OD) at 450 nm was analyzed in a 4 parameter (4PL) nonlinear regression model. Half maximal effective concentration (EC50) was calculated for each individual sample based on duplicate 12-step titration curves. Sample results were expressed as EC50 titers. Values at or below the lower limit of detection (LLOD) were set to the LLOD of 30 EC50. Rabbit serum IgG antibody titers binding to whole *E. coli* cells were evaluated by whole-cell ELISA in 96-well plate format, according to the methods described above. Plates were coated with an equivalent of 0.1 OD_600_ heat inactivated bacterial biomass in PBS per well. Serum samples were added in a 7-step 3-fold serial dilution starting from 1:25000. HRP-labeled goat-anti-rabbit secondary antibody was used at 1:2000 dilution (Southern Biotec cat# 4030-05).

### Opsonophagocytic killing (OPK) assay

Day 42 serum samples from rats immunized with saline or 0.4 µg PS of EPA-Onovel32 or EPA-Onovel32MT- bioconjugates were evaluated for their ability to facilitate opsonophagocytic killing. OPK assays were performed as described before^10^, using representative Onovel32 and Onovel32MT- ExPEC target strains, differentiated HL60 cells and heat inactivated sera added in a 8-step 2-fold serial dilution starting from 1:10. Bacterial killing induced by serum antibodies was analyzed using Protocol 3 colony counter software (v2.1). The number of colonies from duplicate titration curves were determined and the values expressed as Opsonization Index (OI) titers, i.e., the dilution that gives 50% killing of bacteria. Values at or below the LLOD were set to the LLOD of OI 20. Statistical analysis of OI titers was performed in GraphPad Prism v10.1.2 using the non-parametric Mann-Whitney test and a 3-fold Bonferroni correction for multiple testing.

## Supplementary Information

Below is the link to the electronic supplementary material.

Supplementary Material 1

Supplementary Material 2

## Data Availability

Whole genome sequencing data of the *E. coli* isolates analysed in the current study are part of a larger dataset available in the European Nucleotide Archive (ENA) of the European Bioinformatics Institute under accession number PRJEB97262 (https://www.ebi.ac.uk/ena/browser/view/PRJEB97262).
