# Supplementary material for: Molecular epidemiology and structural diversity of O101/O162 O-antigen variants among Escherichia coli bacteremia isolates
Source: Sci Rep. 2026 Mar 23;16:14777. doi: 10.1038/s41598-026-45688-7 (PMC13168486; doi:10.1038/s41598-026-45688-7)
Supplement: Supplementary file 2 — Supplementary Material 2 [file 41598_2026_45688_MOESM2_ESM.docx]

**Supplementary Information**

**Molecular epidemiology and structural diversity of O101/O162 O-antigen variants among *Escherichia coli* bacteremia isolates**

Eveline Weerdenburg^1*^, Mark de Been^1^, Aldert Zomer^2^, Wannisa Ritmahan^1^, Joyce Lübbers^1^, Alan B. Moran^1^, Simone Nicolardi^3^, Manfred Wuhrer^3^, Neil Ravenscroft^4^, Chakkumkal Anish^1a^, Jeroen Geurtsen^1b^, Michel Beurret^1^

^1^ Janssen Vaccines & Prevention B.V., Johnson & Johnson, Leiden, the Netherlands

^2^ Department of Infectious Diseases and Immunology, Faculty of Veterinary Medicine, Utrecht University, Utrecht, the Netherlands

^3^ Center for Proteomics and Metabolomics, Leiden University Medical Center, Leiden, the Netherlands

^4^ Department of Chemistry, University of Cape Town, Rondebosch, South Africa

^a^ Current address: Vaccine & Immunetherapies Division, AstraZeneca, Gaithersburg, MD, USA

^b^ Current address: Sanofi B.V., Amsterdam, The Netherlands

* Corresponding author. E-mail: eveline.weerdenburg@gmail.com

**Supplementary Figures**

**Supplementary Fig. S1**

**Supplementary Fig. S2**

**Supplementary Fig. S3**

**Supplementary Fig. S4**

**Supplementary Fig. S5**


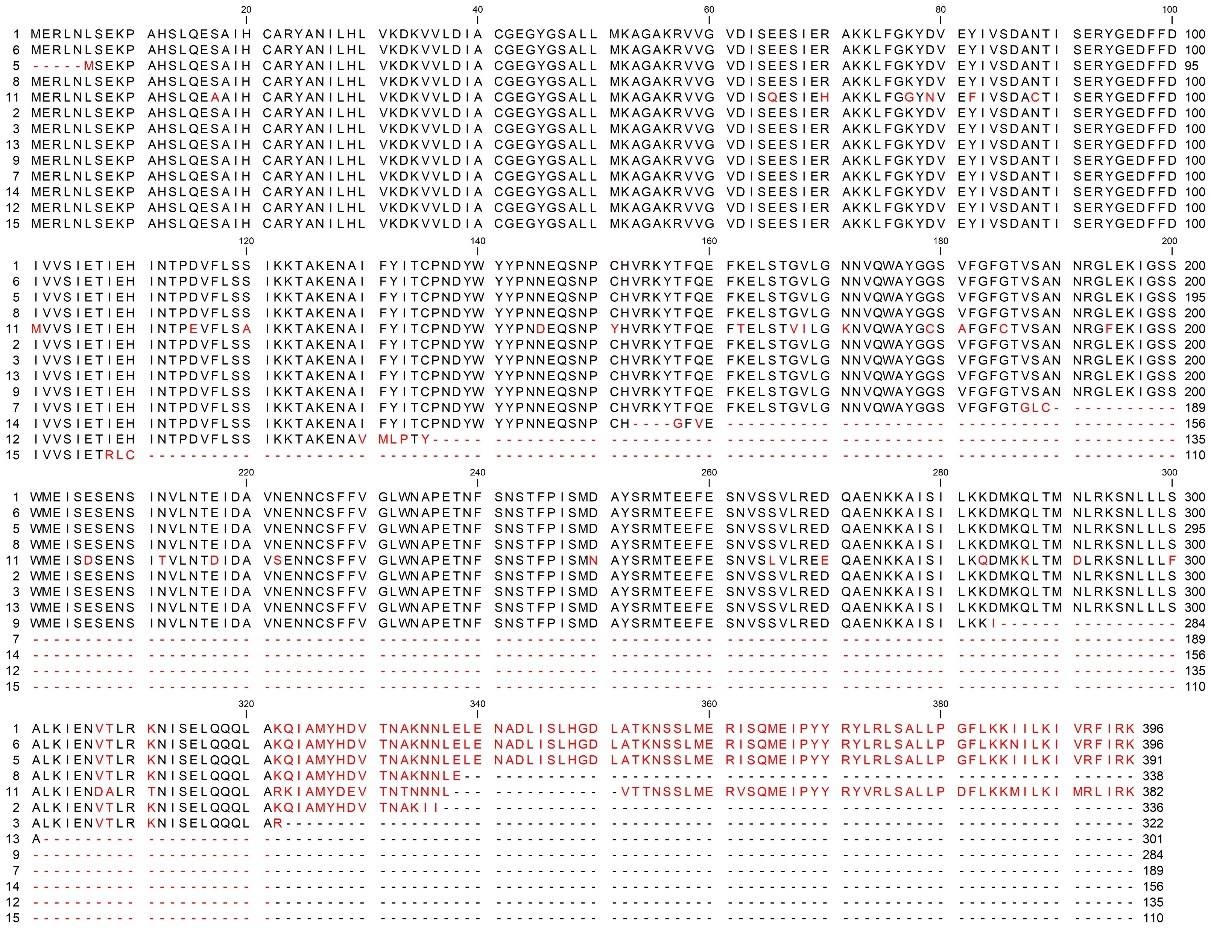


**Supplementary Fig. S1.** Alignment of MT protein sequences identified in Onovel32 *rfb* containing ExPEC isolates. Residues deviating from the consensus sequence are displayed in red. Type 1 represents the intact wild-type sequence that is present in most isolates. For *E. coli* isolates containing type 2 (p.N335fs, BVEC03788), 3 (c.969_970ins[IS1], BVEC01609 and BVEC03285), 8 (p.L339X, BVEC02823), 11 (not shown) and 14 (not shown), an altered LPS profile was confirmed on SDS-PAGE / WB (Supplementary Fig. S3). *E. coli* isolates carrying disrupted MT of other types were not tested for LPS profile on SDS-PAGE / WB.


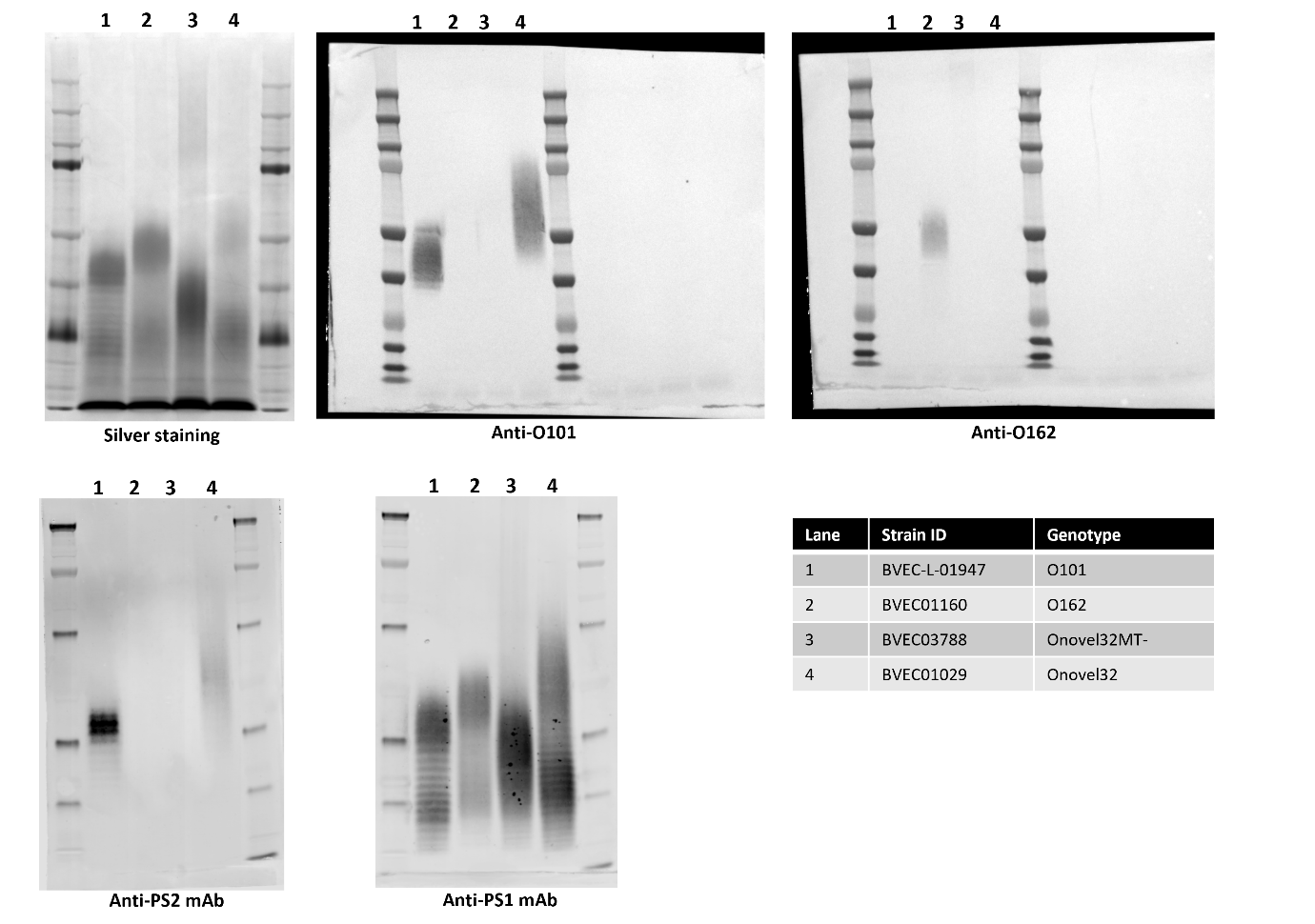


**Supplementary Fig. S2**. Original gel and blot images corresponding to Fig. 4 (top) and Fig. 7 (bottom). Images display silver-stained SDS-PAGE (top left) and Western blots of membranes hybridized with rabbit polyclonal typing serum specific for *E. coli* O101 (top middle) and *E. coli* O162 (top right) or monoclonal antibodies specific for PS2 (bottom left) or PS1 (bottom right), performed on polysaccharide samples of *E. coli* isolates. For Fig. 7 blot images were cropped to display only samples #3 and #4 in panel B, to align with the bioconjugate samples shown in panel A.


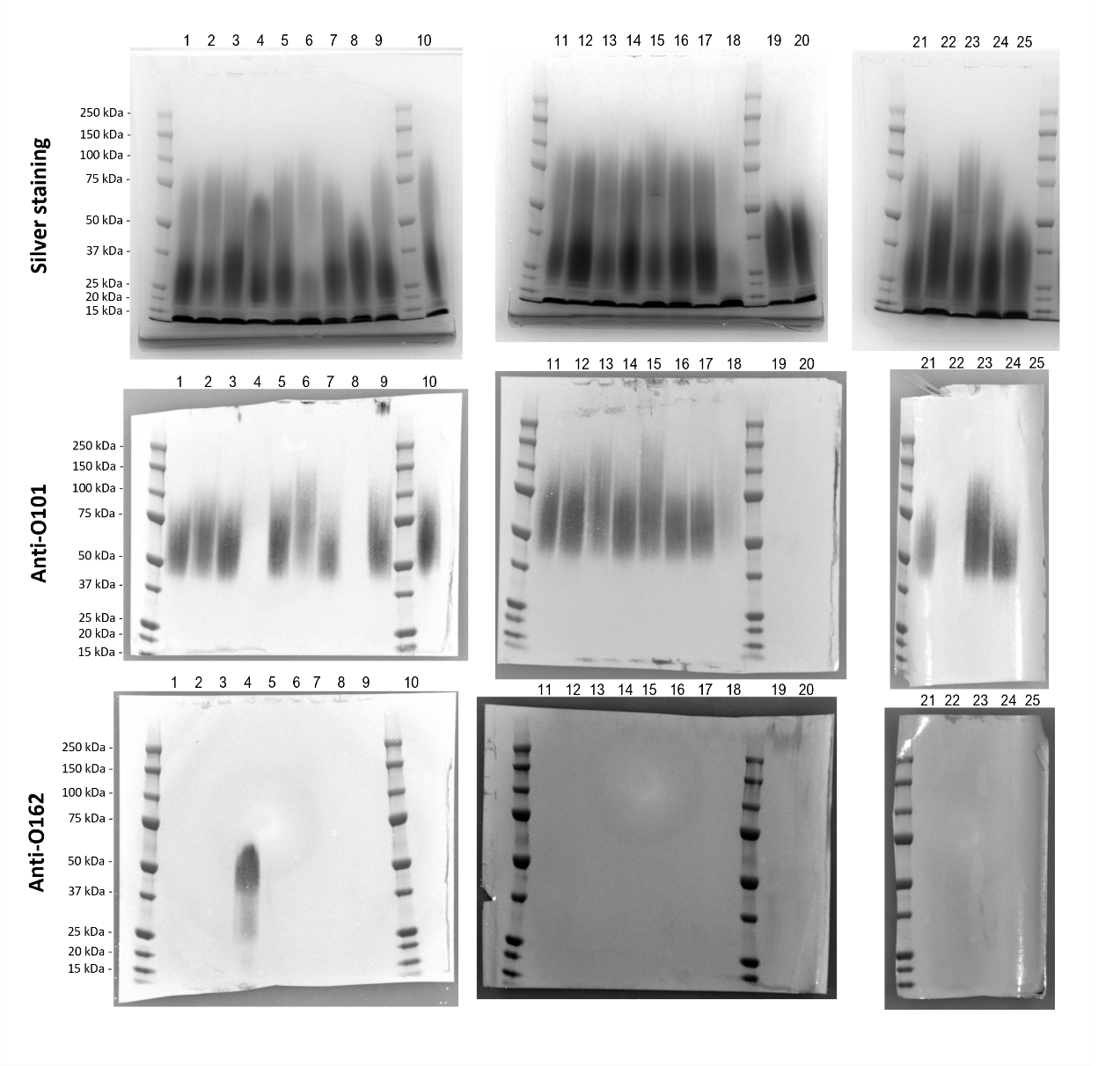


| **Lane** | **Strain ID** | **Agglutination** | ***rfb* locus** | **Sequence deviation Onovel32 *rfb*** |
| --- | --- | --- | --- | --- |
| 1 | BVEC01029 | O101 | Onovel32 | GalE: p.T228A; GT1: p.V475A |
| 2 | BVEC01117 | O101 | Onovel32 | GT1: p.V475A; GT3: p.M297L |
| 3 | BVEC01122 | O162 | Onovel32 | GT1: p.V475A |
| 4 | BVEC01160 | O162 | O162 | N/A |
| 5 | BVEC01606 | O101 | Onovel32 | GT1: p.V475A |
| 6 | BVEC01409 | O101 | Onovel32 | GT3: p.E19K |
| 7 | BVEC01431 | O101 | Onovel32 | - |
| 8 | BVEC01609 | O162 | Onovel32MT- | GT1: p.V475A; MT: c.969_970ins[IS1] |
| 9 | BVEC01735 | O101 | Onovel32 | GT1: p.V475A |
| 10 | BVEC01841 | O101 | Onovel32 | - |
| 11 | BVEC01912 | O101 | Onovel32 | GT1: p.V475A |
| 12 | BVEC01915 | O101 | Onovel32 | GT1: p.V475A |
| 13 | BVEC02075 | O101 | Onovel32 | - |
| 14 | BVEC02213 | O101 | Onovel32 | GT1: p.V475A |
| 15 | BVEC02221 | O101 | Onovel32 | - |
| 16 | BVEC02433 | O101 | Onovel32 | GT1: p.V475A |
| 17 | BVEC02453 | O101 | Onovel32 | GT1: p.V475A |
| 18 | BVEC03828 | O101 | Onovel32 | GT2: p.V69E |
| 19 | BVEC03450 | O162 | Onovel32 | GT1: p.V475A; GT4 p.V497G |
| 20 | BVEC03788 | O162 | Onovel32MT- | GT1: p.V475A; MT: p.N335fs |
| 21 | BVEC03809 | O101 | Onovel32 | GT1: p.V475A |
| 22 | BVEC02823 | O162 | Onovel32MT- | GT1: p.V475A; MT: p.L339X |
| 23 | BVEC02858 | O101 | Onovel32 | - |
| 24 | BVEC03024 | O101 | Onovel32 | GT1: p.V475A |
| 25 | BVEC03285 | O162 | Onovel32MT- | GT1: p.V475A; MT: c.969_970ins[IS1] |

**Supplementary Fig S3**. LPS profile of 25 *E. coli* O101/O162 strains. LPS extracted from *E. coli* O101/O162 blood isolates containing *rfb* loci of O162, Onovel32MT- and Onovel32 was visualized on silver stained SDS-PAGE, Western blot using anti O101 typing serum (SSI cat# 85095) or O162 typing serum (SSI cat #85158). Legend table includes for each isolate the agglutination O-serotype, the identified *rfb* locus and any sequence deviations relative to the Onovel32 reference *rfb* locus resulting in sequence variation at the protein level.


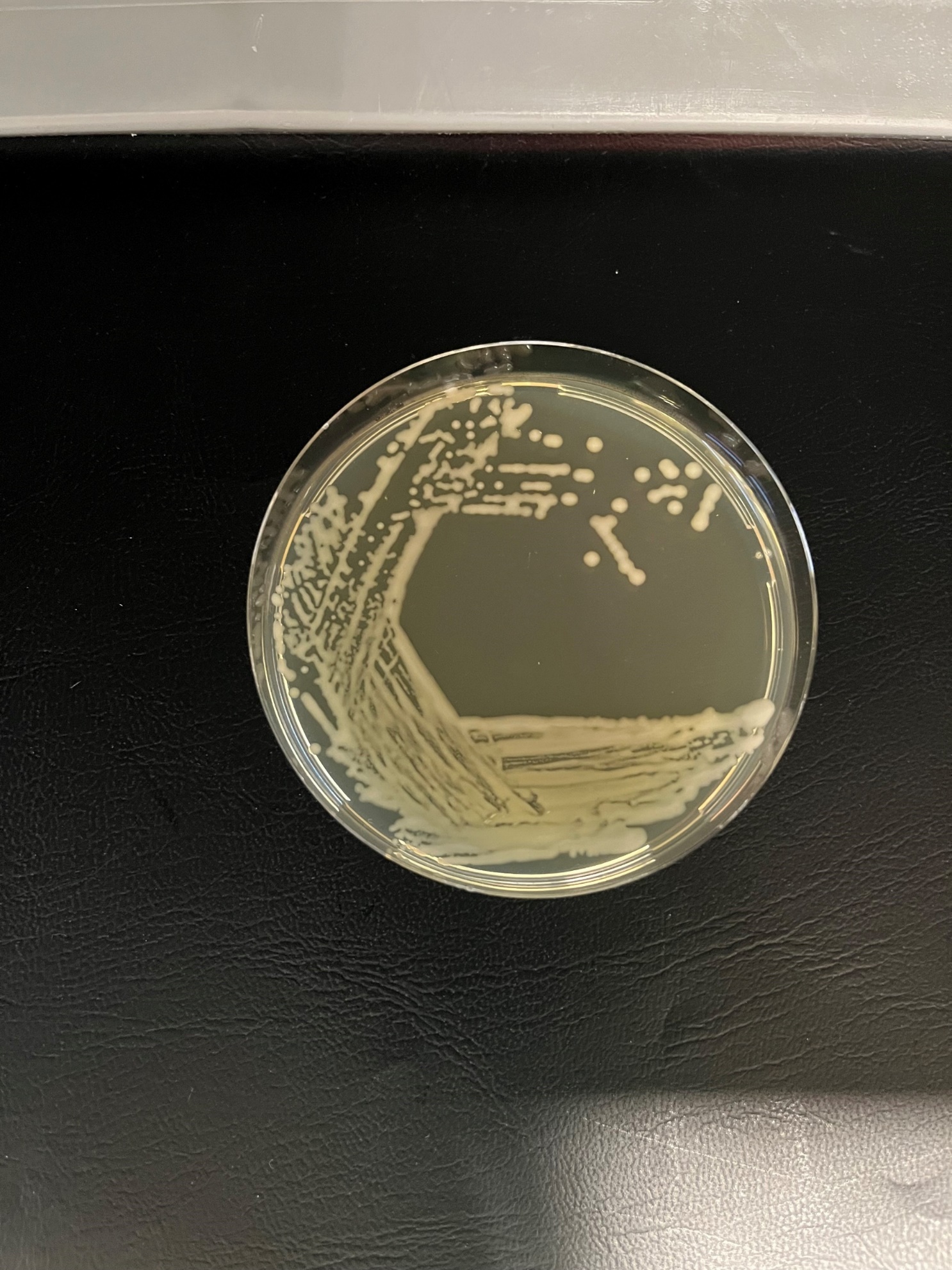


**Supplementary Fig. S4**. Image showing a representative Onovel32 *rfb* containing ExPEC isolate with a mucoviscous phenotype, as observed by its wet, slimy appearance on an LB agar plate.

**A**

**B**

**EPA-Onovel32**

**EPA-Onovel32MT-**

**Supplementary Fig. S5**. EC50 titers against *E. coli* O101/O162 O-serogroup strains measured in serum from rabbits immunized with **(A)** EPA-Onovel32 or **(B)** EPA-Onovel32MT- bioconjugate and CFA/IFA adjuvant in a whole-cell ELISA assay. Plates were coated with biomass from *E. coli* strains of O101/O162 O-serogroup subtypes O101 (n=1), O162 (n=2), Onovel32 (n=21) and Onovel32MT- (n=9). In addition, *E. coli* control strains of O-serotype O9 (n=1) and rough strains (NEG, n=2) were evaluated for background titers.
